# Genetic and Metabolomic Dissection of the Ergothioneine and Selenoneine Biosynthetic Pathway in the Fission Yeast, *S. pombe*, and Construction of an Overproduction System

**DOI:** 10.1371/journal.pone.0097774

**Published:** 2014-05-14

**Authors:** Tomáš Pluskal, Masaru Ueno, Mitsuhiro Yanagida

**Affiliations:** 1 G0 Cell Unit, Okinawa Institute of Science and Technology Graduate University (OIST), Onna-son, Okinawa, Japan; 2 Department of Molecular Biotechnology, Graduate School of Advanced Sciences of Matter, Hiroshima University, Higashihiroshima-shi, Hiroshima, Japan; Cancer Research UK London Research Institute, United Kingdom

## Abstract

Ergothioneine is a small, sulfur-containing metabolite (229 Da) synthesized by various species of bacteria and fungi, which can accumulate to millimolar levels in tissues or cells (e.g. erythrocytes) of higher eukaryotes. It is commonly marketed as a dietary supplement due to its proposed protective and antioxidative functions. In this study we report the genes forming the two-step ergothioneine biosynthetic pathway in the fission yeast, *Schizosaccharomyces pombe*. We identified the first gene, *egt1^+^* (SPBC1604.01), by sequence homology to previously published genes from *Neurospora crassa* and *Mycobacterium smegmatis*. We showed, using metabolomic analysis, that the Δ*egt1* deletion mutant completely lacked ergothioneine and its precursors (trimethyl histidine/hercynine and hercynylcysteine sulfoxide). Since the second step of ergothioneine biosynthesis has not been characterized in eukaryotes, we examined four putative homologs (Nfs1/SPBC21D10.11c, SPAC11D3.10, SPCC777.03c, and SPBC660.12c) of the corresponding mycobacterial enzyme EgtE. Among deletion mutants of these genes, only one (Δ*SPBC660.12c*, designated Δ*egt2*) showed a substantial decrease in ergothioneine, accompanied by accumulation of its immediate precursor, hercynylcysteine sulfoxide. Ergothioneine-deficient strains exhibited no phenotypic defects during vegetative growth or quiescence. To effectively study the role of ergothioneine, we constructed an *egt1^+^* overexpression system by replacing its native promoter with the *nmt1^+^* promoter, which is inducible in the absence of thiamine. We employed three versions of the *nmt1* promoter with increasing strength of expression and confirmed corresponding accumulations of ergothioneine. We quantified the intracellular concentration of ergothioneine in *S. pombe* (0.3, 157.4, 41.6, and up to 1606.3 µM in vegetative, nitrogen-starved, glucose-starved, and *egt1^+^*-overexpressing cells, respectively) and described its gradual accumulation under long-term quiescence. Finally, we demonstrated that the ergothioneine pathway can also synthesize selenoneine, a selenium-containing derivative of ergothioneine, when the culture medium is supplemented with selenium. We further found that selenoneine biosynthesis involves a novel intermediate compound, hercynylselenocysteine.

## Introduction

Ergothioneine (EGT) is a sulfur-containing *Nα,Nα,Nα*-trimethyl-L-histidine-derived metabolite that is synthesized by various species of bacteria and fungi (recently extensively reviewed by Cheah and Halliwell [1]). Higher organisms obtain EGT in food and accumulate it in certain tissues up to millimolar levels [2], [3] through a specific transporter, ETT/OCTN1 [4]. In mammals, large amounts of EGT are found in erythrocytes, bone marrow, liver, kidney, eye lens, and seminal fluid [2], [5], [6]. Nevertheless, EGT is neither a nutrient (it is virtually unmetabolized in humans) nor a vitamin (it is non-essential). EGT is commonly marketed as a dietary supplement or nutraceutical, due to its anti-oxidant properties *in vitro*, reported in numerous publications [3], [7]–[10]. Direct scavenging of free radicals and chelation of transition metals are the most widely cited possible functions of EGT [10], [11]. However, so far, no rigorous research has conclusively demonstrated any benefit of EGT *in vivo*. It is unclear whether EGT consumption contributes to human health, and if it does, what daily intake is optimal. It is thus of considerable interest for biologists and medical scientists to uncover the physiological mechanism of EGT at the molecular level. Recently, biosynthetic pathways for EGT have been characterized in *Mycobacterium smegmatis* [12] and *Neurospora crassa* [13], allowing the use of genetic methods.

The fission yeast, *Schizosaccharomyces pombe*, is a suitable model organism for the study of cell division and quiescence [14]–[16]. We have previously established a method of comprehensive metabolomic analysis in *S. pombe* using liquid chromatography-mass spectrometry (LC-MS) [17]. Among the several hundred observed metabolites, we also identified EGT and described its accumulation under glucose starvation [18] and nitrogen starvation [19]. In addition, we reported abnormally high accumulations of EGT in the proteasome regulatory subunit mutant *mts3-1*, which suffers from severe oxidative stress caused by mitochondrial dysfunctions in G0 arrest [20], and in the Krüppel-like zinc-finger transcription factor deletion mutant Δ*klf1*, which exhibits cell wall defects in long-term quiescence, accompanied by up-regulation of mitochondrial transcripts [21]. Interestingly, EGT is not synthesized by the budding yeast, *Saccharomyces cerevisiae* [22], suggesting that not all fungi require EGT for normal cellular function. Due to its simplicity and advanced genetics, *S. pombe* might represent an ideal unicellular, eukaryotic system to study the biochemical role of EGT.

Here we report identification of genes forming the two-step biosynthetic pathway of EGT from histidine in *S. pombe*, through the combined use of genetic and metabolomic approaches. Among other compounds, we were able to identify the direct precursor of EGT, hercynylcysteine sulfoxide. We constructed an EGT and hercynylcysteine sulfoxide overproduction system utilizing three different overexpression strains with the inducible *nmt1^+^* promoter. We quantified intracellular EGT content in wild type (WT) as well as overexpression cells by constructing a calibration curve from pure EGT standard injections into the LC-MS. Further, we show the accumulation of EGT under long-term quiescence. Finally, we demonstrate that the EGT pathway can also synthesize selenoneine, a selenium-containing derivative of EGT, the production of which involves a novel intermediate compound, hercynylselenocysteine.

## Results

### EGT biosynthesis pathway

**Figure 1:**
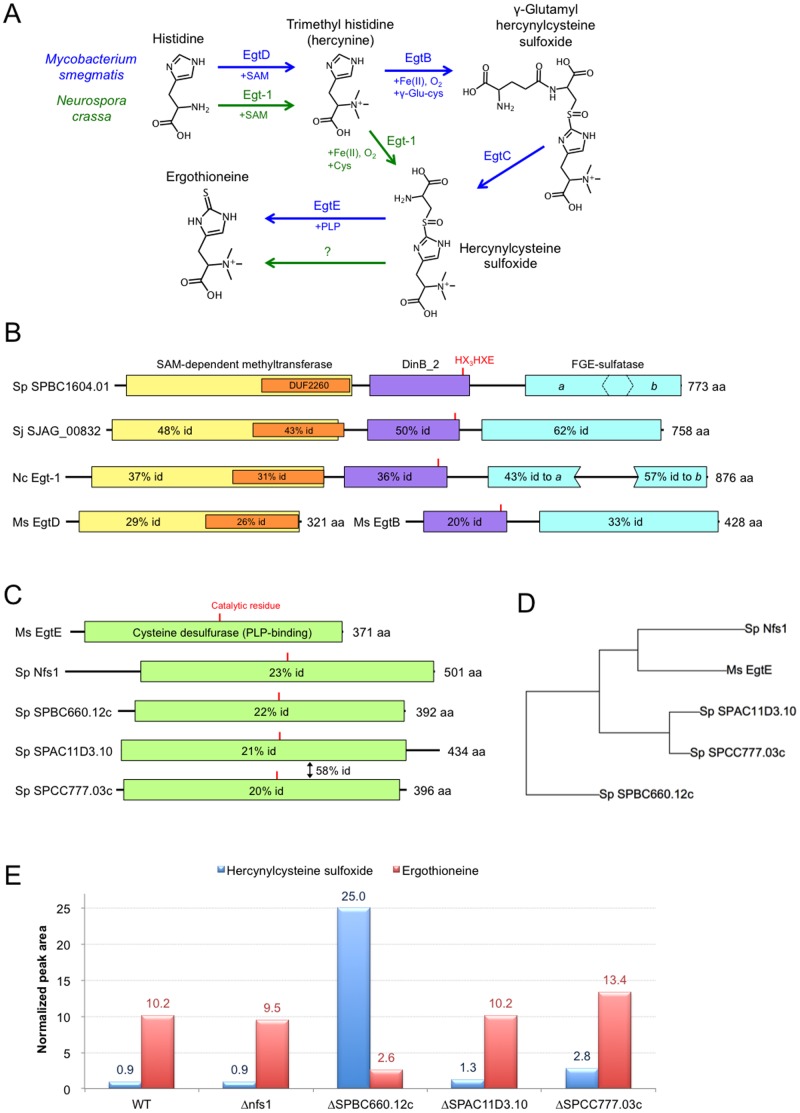
Characterization of EGT biosynthesis in *S. pombe*. **A.** Previously published EGT biosynthesis pathways in *M. smegmatis* and *N. crassa*. The Egt-1 protein in *N. crassa* is a fusion enzyme that catalyzes two different reactions. **B.** Comparison of the conserved domain structure of *S. pombe* protein SPBC1604.01 (designated Egt1 in this manuscript) with *S. japonicus* SJAG_00832 and EGT biosynthesis proteins in *N. crassa* (Egt-1) and *M. smegmatis* (EgtD and EgtB). All proteins are composed of: 1. an S-adenosyl-L-methionone (SAM)-dependent methyltransferase domain, including DUF2260, a domain of unknown function; 2. an uncharacterized DinB_2 domain, including an iron-binding motif HX_3_HXE; and 3. a formylglycine generating enzyme (FGE)-sulfatase domain. Percentage identity (% id) of amino acid sequences is indicated in comparison to the corresponding sequence in *S. pombe*. **C.** Comparison of conserved domain structure of *M. smegmatis* protein EgtE with its four putative homologs in *S. pombe*. All proteins contain a single pyridoxal phosphate (PLP)-binding cysteine desulfurase domain. The conserved catalytic residues (PLP binding sites) are indicated by red lines. Percentage identity (% id) of the amino acid sequences is indicated in comparison to the corresponding sequence in *M. smegmatis*. **D.** Phylogenetic tree visualizing the similarity of amino acid sequences of *M. smegmatis* EgtE and its putative homologs in *S. pombe*. **E.** Normalized peak areas of hercynylcysteine sulfoxide and EGT obtained by metabolomic analysis of WT and deletion mutant *S. pombe* strains. Cells were nitrogen-starved prior to analysis (24 h in EMM2-N medium) to induce EGT synthesis.

The reported EGT biosynthetic pathways in *M. smegmatis* and *N. crassa* are schematized in Figure 1A. On the basis of sequence homology, the *S. pombe* locus, *mug158^+^*/SPBC1604.01, was previously suggested to encode the main EGT biosynthetic enzyme [12], [13]. This enzyme, with 773 amino acids, catalyzes triple methylation of histidine to hercynine (*Nα,Nα,Nα*-trimethyl histidine) and subsequent conjugation with cysteine and oxygen to form hercynylcysteine sulfoxide. *mug158^+^*/SPBC1604.01 is a distant homolog of the mycobacterial EgtD and EgtB genes, encoding a single fusion protein. Figure 1B shows the domain structure of this protein, according to the Conserved Domain Database [23], in comparison with its homologs in *Schizosaccharomyces japonicus* (a relative of *S. pombe*), *N. crassa*, and *M. smegmatis*. The exact locations and sequences of individual domains are shown in Table S1 and the amino acid sequence alignment is shown in Figure S1. Due to its structure and sequence similarities to NcEgt-1, we presume that the *S. pombe* homolog also utilizes cysteine as a substrate, rather than using γ-glutamyl-cysteine, as in the case of the bacterial EgtB enzyme. The bacterial pathway requires a subsequent removal of the glutamyl residue by another enzyme, EgtC [12], which has no obvious homolog in the genome of *S. pombe*. Since the *mug158^+^*/SPBC1604.01 gene encodes the first step of EGT synthesis, we hereafter designate it *egt1^+^*.

The final step of EGT biosynthesis in bacteria is represented by EgtE, a pyridoxal phosphate (PLP)-binding cysteine desulfurase that forms the end product, EGT, from hercynylcysteine sulfoxide, by cleaving the cysteine residue at the sulfur atom. To date, the EgtE homolog has not been characterized in *N. crassa*. The *S. pombe* genome contains four cysteine desulfurases that could be homologous to EgtE: Nfs1 (SPBC21D10.11c), SPBC660.12c, SPAC11D3.10, and SPCC777.03c (Figure 1C; location and sequences of conserved domains in Table S2; amino acid sequence alignment in Figure S2). Among these, SPAC11D3.10, and SPCC777.03c have highly similar sequences, possibly originating from horizontal gene transfer (Figure 1D).

To identify the EgtE homolog in *S. pombe*, we obtained deletion mutants from the Bioneer haploid deletion library [24], cultivated them under nitrogen starvation (EMM2 medium lacking NH_4_Cl, hereafter designated EMM2-N) for 24 hours to induce EGT production, and performed a metabolomic analysis. Among the four deletion mutants tested, Δ*SPBC660.12c* was the only one showing a substantial decrease in EGT and an increase in its precursor, hercynylcysteine sulfoxide (Figure 1E; numerical results of all LC-MS measurements shown in Table S3). We thus designated the SPBC660.12c locus *egt2^+^*, as it represents the gene primarily responsible for the second step of EGT biosynthesis in *S. pombe*.

### Verification of *egt1^+^* and *egt2^+^* by metabolomic analysis

To verify correct assignment of the *egt1^+^* and *egt2^+^* genes, we newly constructed full deletion mutants, Δ*egt1* and Δ*egt2*, by replacing the target loci in the WT *h^−^* 972 strain with the kanamycin resistance marker (kanMX). Correct integration of the kanMX modules into the new strains was verified by PCR. No difference from WT in cell size or shape was observed in these strains under any of the analyzed conditions. Table 1 shows the results of metabolome analysis of the constructed strains under starvation (EMM2-N or EMM2 medium with low concentration of glucose, 1.1 mM, hereafter designated EMM2-LG, for 24 hours). Data regarding EGT and its precursors, starting with histidine, are shown. We confirmed the absence of all pathway intermediates in Δ*egt1*, and the accumulation of hercynylcysteine sulfoxide in Δ*egt2*. A small amount of EGT was still found in Δ*egt2* under starvation. To determine whether any other cysteine desulfurase might contribute to this enzymatic reaction, we constructed multiple deletion mutants of SPBC660.12c (*egt2^+^*) with the other candidate EgtE homologs, but even in successfully constructed double and triple mutants, a significant amount of EGT still remained (Figure S3). This, however, is not a surprising result, as Seebeck [12] previously demonstrated that hercynylcysteine sulfoxide could spontaneously convert into EGT in the presence of PLP. Furthermore, as also shown by Seebeck [12], this reaction could be catalyzed by an unrelated PLP-binding β-lyase originating from *Erwinia tasmaniensis*. Thus, we suspect the residual EGT found in Δ*egt2* might be a product of a hercynylcysteine sulfoxide reaction with PLP, possibly catalyzed by an unrelated PLP-binding enzyme (*S. pombe* genome contains at least 26 PLP-binding enzymes, according to PomBase [25]).

**Table 1:** Normalized peak areas of the four compounds composing the EGT biosynthetic pathway obtained by metabolomic analysis of WT and newly constructed strains.

Compound, peak m/z and retention time/Strain,		Histidine	Trimethyl-histidine (hercynine)	Hercynyl-cysteine sulfoxide	Ergothio-neine
cultivation condition		156.077 m/z @12.4 min	198.124 m/z @10.3 min	333.123 m/z @12.2 min	230.096 m/z @12.6 min
	EMM2	14.4	1.7	0	0.1
WT 972	EMM2-N (24 h)	2.2	3.2	2.2	13.7
	EMM2-LG (24 h)	55.4	65.9	3.5	6.7
	EMM2	10.7	0.3	0	0
Δ*egt1*	EMM2-N (24 h)	2.8	0.1	0	0
	EMM2-LG (24 h)	54.1	0	0	0
	EMM2	11.4	0.9	3.9	0
Δ*egt2*	EMM2-N (24 h)	1.9	1.8	58.2	3.1
	EMM2-LG (24 h)	61.1	64.7	44.1	1.6

Values were measured from metabolome samples of four different *S. pombe* strains in three different cultivation conditions, as indicated. Mass values (m/z) and LC retention times (min) of each peak are included for reference.

**Figure 2:**
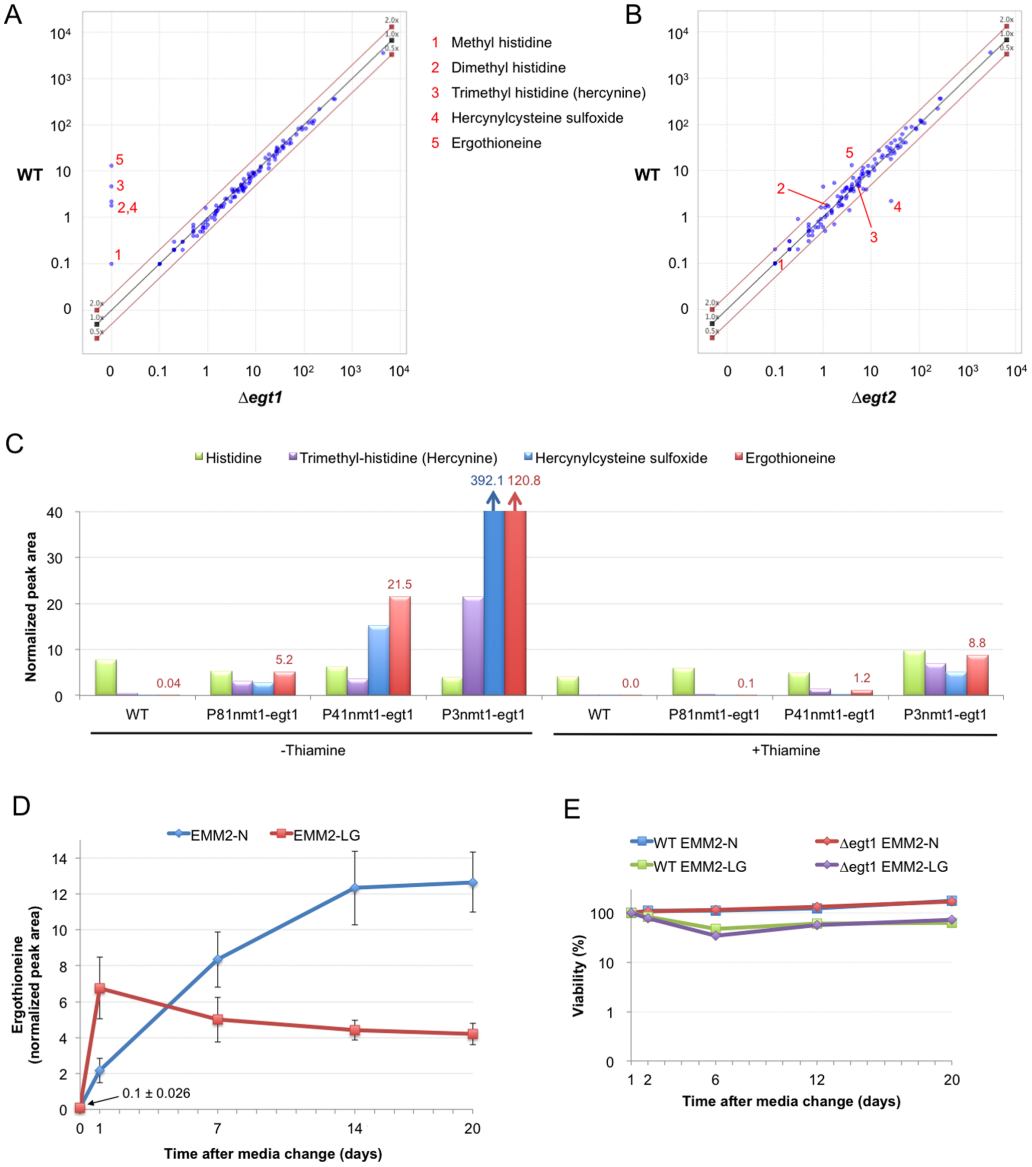
Characterization of Δ*egt1* and Δ*egt2* strains. **A and B.** A scatter plot comparing results of metabolomic analysis of WT vs. Δ*egt1* (A) or Δ*egt2* (B) strains under nitrogen starvation (24 h in EMM2-N medium). Each dot represents a single identified metabolite. Values on both scales indicate normalized peak areas of metabolite peaks in corresponding strains. Red diagonal lines indicate a 2-fold difference. **C.** Results of metabolomic analysis of WT and *egt1^+^* overexpression strains. Cells were cultivated at 26°C in the EMM2 medium lacking thiamine for at least 24 h. Cultures indicated +Thiamine were cultivated in the presence of 5 µg/ml thiamine for 24 h. Normalized peak areas of compounds composing the EGT pathway are shown. **D.** Time course metabolomic analysis of quiescent *S. pombe* cultures under nitrogen (EMM2-N) and glucose (EMM2-LG) starvation. Values represent means ± standard deviations of normalized peak areas of EGT in three independent cell cultures. **E.** Time course viability results of WT and Δ*egt1* deletion mutant cultures under nitrogen (EMM2-N) and glucose (EMM2-LG) starvation.

Neither the Δ*egt1* nor the Δ*egt2* strain showed any growth defects during cultivation in either rich (YE) or minimal (EMM2) culture media. Furthermore, deletion of *egt1^+^* or *egt2^+^* caused no significant perturbation to the intracellular metabolome of quiescent cells (Figures 2A and 2B). Apart from the disappearance of EGT and its precursors, all other metabolite levels remained within the range of common experimental error.

### Conservation of *egt1^+^* and *egt2^+^* in other species

As observed by Seebeck [12], the EGT biosynthestic pathway can be found in a relatively small number of eukaryotes, notably in the phyla *Basidiomycota* (most species) and *Ascomycota* (most species in subphyla *Pezizomycotina* and *Schizosaccharomycetes*). We summarized homologs of EGT biosynthesis genes in several organisms closely related to *S. pombe* (Table 2). The exact locations of the conserved domains in these enzymes are shown in Tables S4 and S5. According to sequence alignment (Figures S4 and S5), conserved domains show higher homology among species than inter-domain regions. Methyltransferase and FGE-sulfatase domains contain long non-homologous sequence inserts in *U. maydis* UM00197. The function of the inserts in these two domains is unknown, however, they are not unusual among various species [13].

**Table 2:** Closest homologs of *S. pombe* Egt1 and Egt2 proteins in selected species.

Organism	Closest homolog of *S. pombe* Egt1	Closest homolog of *S. pombe* Egt2
*Schizosaccharomyces japonicus*	SJAG_00832	SJAG_03856
*Schizosaccharomyces octosporus*	SOCG_01424	SOCG_02548
*Neurospora crassa*	NCU04343 (NcEgt-1)	NCU11365
*Aspergillus niger*	An15g05880	An02g02030 or An05g02190
*Aspergillus oryzae*	Ao090012000265	Ao090026000291
*Ustilago maydis*	UM00197	UM04128

Candidate homologs were searched using the on-line version of the Basic Local Alignment Search Tool (http://blast.ncbi.nlm.nih.gov) for protein sequences (blastp) and candidates with the best similarity scores (lowest blastp E-values) were selected.

Based on sequence homology to *S. pombe* Egt2 (33% amino acid sequence identity), we propose that the NCU11365 gene may encode the second enzyme for EGT biosynthesis in *N. crassa*. Bello et al. [13] previously suggested the NCU04636 gene (19% identity to Egt2), which appears to correspond to *nfs1* ^+^, the mitochondrial cysteine desulfurase in *S. pombe* (63% identity). Both *S. pombe nfs1* ^+^ and *N. crassa* NCU04636 appear to be the closest homologs of mycobacterial EgtE (as also indicated in Figure 1D). However, we did not detect any change in hercynylcysteine sulfoxide levels in the Δ*nfs1* deletion mutant (Figure 1E). Furthermore, the *nfs1* ^+^ gene has apparent homologs in species that do not produce EGT, such as *S. cerevisiae* or humans (both genes called NFS1).

### Overexpression of *egt1^+^*

To study the overproduction effect of EGT in *S. pombe*, we applied the method of Bähler et al. [26] to replace the *egt1^+^* native promoter with the *nmt1^+^* promoter, which is inducible in the absence of thiamine [27]. We employed three versions of the *nmt1* promoter plasmid with increasing strength of expression and constructed three strains *P81nmt1-egt1^+^*, *P41nmt1-egt1^+^*, and *P3nmt1-egt1^+^*, respectively. Using metabolomic analysis we confirmed the accumulation of EGT and its precursors in these strains, and this accumulation was effectively suppressed by the addition of 5 µg/ml thiamine to the EMM2 medium (Figure 2C).

### Intracellular EGT content

Wild type *S. pombe* cells contain only trace amounts of EGT under normal vegetative conditions (Table 1). However, EGT increases in quiescent cells under starvation [18], [19]. We measured the areas of EGT peaks under vegetative, quiescent, and *egt1^+^*-overexpressing conditions, and converted them to absolute concentrations using a calibration curve based upon pure EGT injections ranging from 1 fmol - 10 nmol (Figure S6). Intracellular volume was assumed to be 148.5 µm^3^ for vegetative cells [28]. For nitrogen- and glucose-starved cells, intracellular volumes were estimated as 1/3 and 2/3 of the vegetative cell volume, respectively. The resulting intracellular concentrations are shown in Table 3.

**Table 3:** Absolute intracellular EGT concentrations ( µM) in *S. pombe* cells.

Cell condition	Culture medium	Intracellular EGT ( µM)
WT vegetative	EMM2	0.3
WT nitrogen starvation	EMM2-N (24 h)	157.4
WT glucose starvation	EMM2-LG (24 h)	41.6
*P81nmt1-egt1^+^*	EMM2	32.4
*P41nmt1-egt1^+^*	EMM2	181.2
*P3nmt1-egt1^+^*	EMM2	1606.3

Intracellular concentrations were derived from measured normalized peak areas using a calibration curve generated by injections of pure EGT in 10-fold dilution steps. The detailed calculation method is described in Figure S6.

To assess long-term variation in EGT content, we measured the level of intracellular EGT using metabolomic analysis during a 20-day time course under quiescence induced by nitrogen (EMM2-N) and glucose (EMM2-LG) starvation, respectively (Figure 2D). Cells showed time-dependent accumulation of EGT, despite being deprived of nutrients, suggesting that EGT might support cellular health under long-term quiescence. However, no loss of viability was observed in Δ*egt1* mutant during 20 days of starvation (Figure 2E).

### Contribution of *egt1^+^* to oxidative stress response

As EGT is generally considered to be a physiological antioxidant, and the Δ*NcEgt-1* mutant was reportedly sensitive to *tert*-butyl hydroperoxide in *N. crassa* [13], we performed spot test experiments on EMM2 plates containing oxidants hydrogen peroxide and *tert*-butyl hydroperoxide, using the deletion and overexpression mutants described above (Figure S7). However, we did not observe any sensitivity or resistance of these strains compared to WT 972 strain, suggesting that *egt1^+^* might not be among the primary mechanisms that protect *S. pombe* from exogenous peroxide.

### Biosynthesis of selenoneine

**Figure 3:**
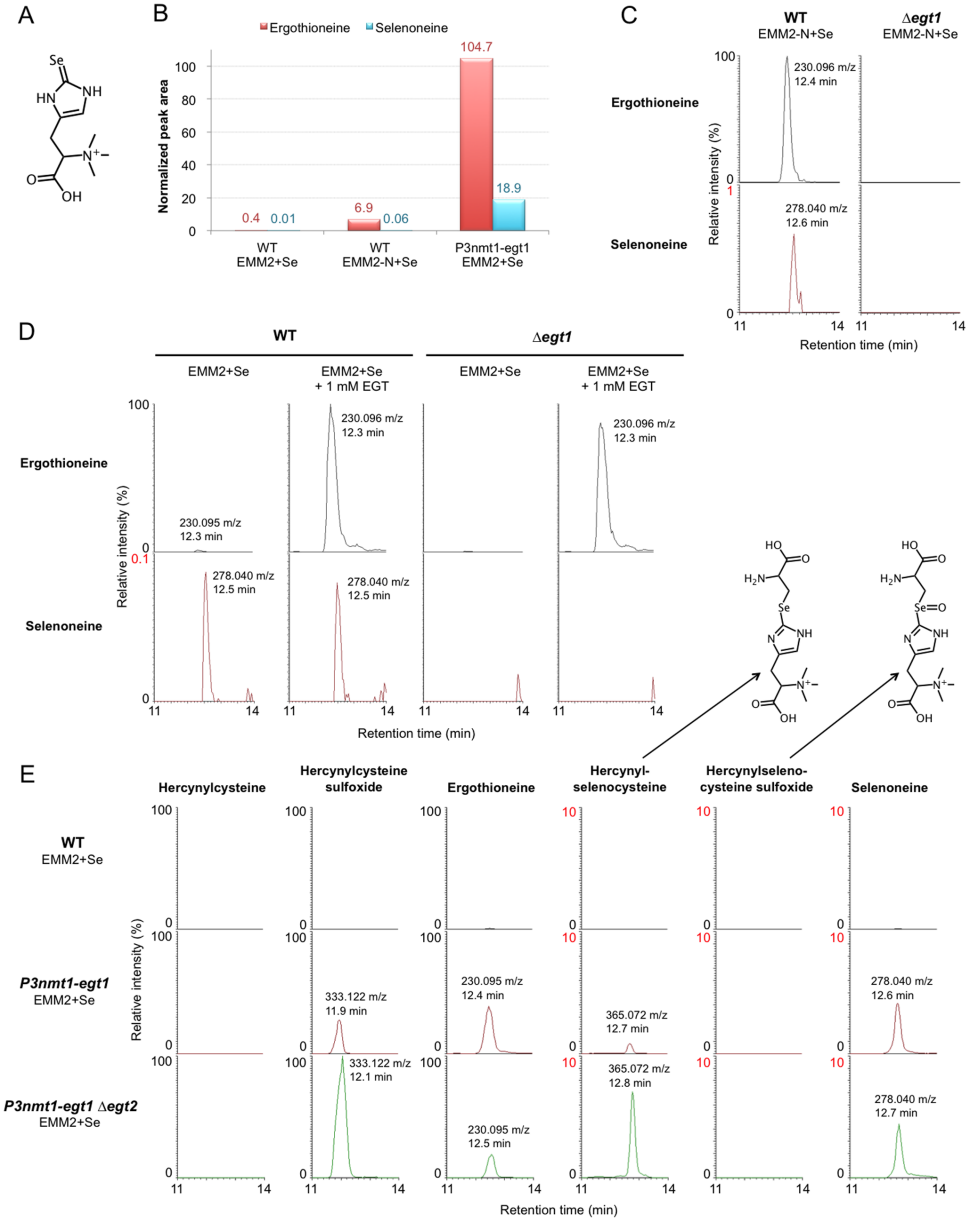
Production of selenoneine in *S. pombe*. **A.** Chemical structure of selenoneine. **B.** Results of metabolomic analysis of WT and *P3nmt1-egt1^+^* strains in EMM2+Se and EMM2-N+Se media. Normalized peak areas of EGT and selenoneine are shown. **C.** Extracted ion chromatograms of EGT and selenoneine masses in raw LC-MS data acquired from metabolome samples of nitrogen-starved cells (24 h in EMM2-N+Se medium) of WT and Δ*egt1* strains. Note that the intensity scale of the selenoneine plot is 1% relative to that of the EGT plot. **D.** Extracted ion chromatograms of EGT and selenoneine masses in raw LC-MS data acquired from metabolome samples of WT and Δ*egt1* strains cultivated in EMM2+Se medium with and without supplementation with 1 mM pure EGT. Note that the intensity scale of the selenoneine plot is 0.1% relative to that of the EGT plot. **E.** Extracted ion chromatograms of six compound masses in WT, *P3nmt1-egt1^+^*, and *P3nmt1-egt1^+^ Δegt2* strains. The plots of hercynylcysteine and hercynylselenocysteine sulfoxide show mass values calculated from their predicted chemical formulas (C_12_H_21_N_4_O_4_S^+^  = 317.128 m/z for hercynylcysteine, and C_12_H_21_N_4_O_5_Se^+^  = 381.067 m/z for hercynylselenocysteine sulfoxide, respectively). Note that the intensity scale of the plots in the right half of the figure is adjusted to 10% relative to the plots in the left half of the figure.

Selenoneine (Figure 3A) is a selenium-containing derivative of EGT found in tuna, and possibly implicated in methylmercury detoxification [29], [30]. As the EMM2 medium does not normally contain selenium, it is not surprising that no selenoneine was detected in previous *S. pombe* metabolome data sets. To test whether *S. pombe* can produce selenoneine, we first examined cell cultivation in a liquid EMM2 medium supplemented with various concentrations of Na_2_SeO_4_ (Figure S8). The medium with 10 µM Na_2_SeO_4_ (hereafter designated EMM2+Se) was selected for further experiments, as *S. pombe* cells exhibited quite normal (albeit slightly slower) proliferation in this condition. Metabolomic analysis was performed on WT vegetative cells, WT nitrogen-starved cells (24 h in EMM2-N+Se), and *P3nmt1-egt1^+^* overexpression mutant cells. We could clearly observe accumulation of selenoneine in the *P3nmt1-egt1^+^* strain cultivated in EMM2+Se medium (Figure 3B), suggesting that the overexpressed *egt1^+^* gene was also responsible for selenoneine synthesis. A tiny signal of selenoneine (<1% of the EGT signal intensity) could also be detected in WT nitrogen-starved cells, and the signals of both EGT and selenoneine disappeared in the Δ*egt1* deletion mutant (Figure 3C).

To rule out the possibility that selenoneine was produced by direct conversion from ergothioneine (without requiring any *egt1^+^* activity), we performed two additional experiments. First, pure EGT was mixed with an equal concentration of Na_2_SeO_4_ *in vitro* and incubated at room temperature for 24 h. No selenoneine signal was detected in this mixture (Figure S9). In the second experiment we supplemented the Δ*egt1* mutant with 1 mM EGT, which was apparently transported into the cells and produced a strong EGT peak. No selenoneine was detected in this case either (Figure 3D). Furthermore, the weak, but clearly detectable selenoneine signal found in WT cells did not increase as a result of EGT supplementation. These results suggest that *egt1^+^* activity is indispensable for selenoneine biosynthesis in *S. pombe*.

As we did not observe any signal of the presumed intermediate, hercynyl-selenocysteine sulfoxide, we constructed a double mutant of *P3nmt1-egt1^+^* and Δ*egt2*. This mutant should accumulate large amounts of the intermediate, assuming that the Egt1/Egt2 pathway is used for selenoneine synthesis. Surprisingly, we found a strong signal of hercynylselenocysteine (not sulfoxide) in this mutant (Figure 3E). This signal was also found in the *P3nmt1-egt1^+^* single mutant, but further increased in the double mutant with Δ*egt2*. We thus conclude that selenoneine biosynthesis, unlike EGT biosynthesis, does not produce a sulfoxide as its intermediate, but produces hercynylselenocysteine instead.

Since selenium naturally occurs in a very characteristic set of isotopes, we verified the identities of selenoneine and hercynylselenocysteine peaks by checking their isotope distribution patterns (Figure S10). Finally, to check whether the EGT/selenoneine pathway could possibly be involved in selenium detoxification in *S. pombe*, we performed a spot test experiment on EMM2 plates containing various concentrations of Na_2_SeO_4_. However, none of the analyzed mutants showed any growth differences compared with WT cells (Figure S11).

## Discussion

**Figure 4:**
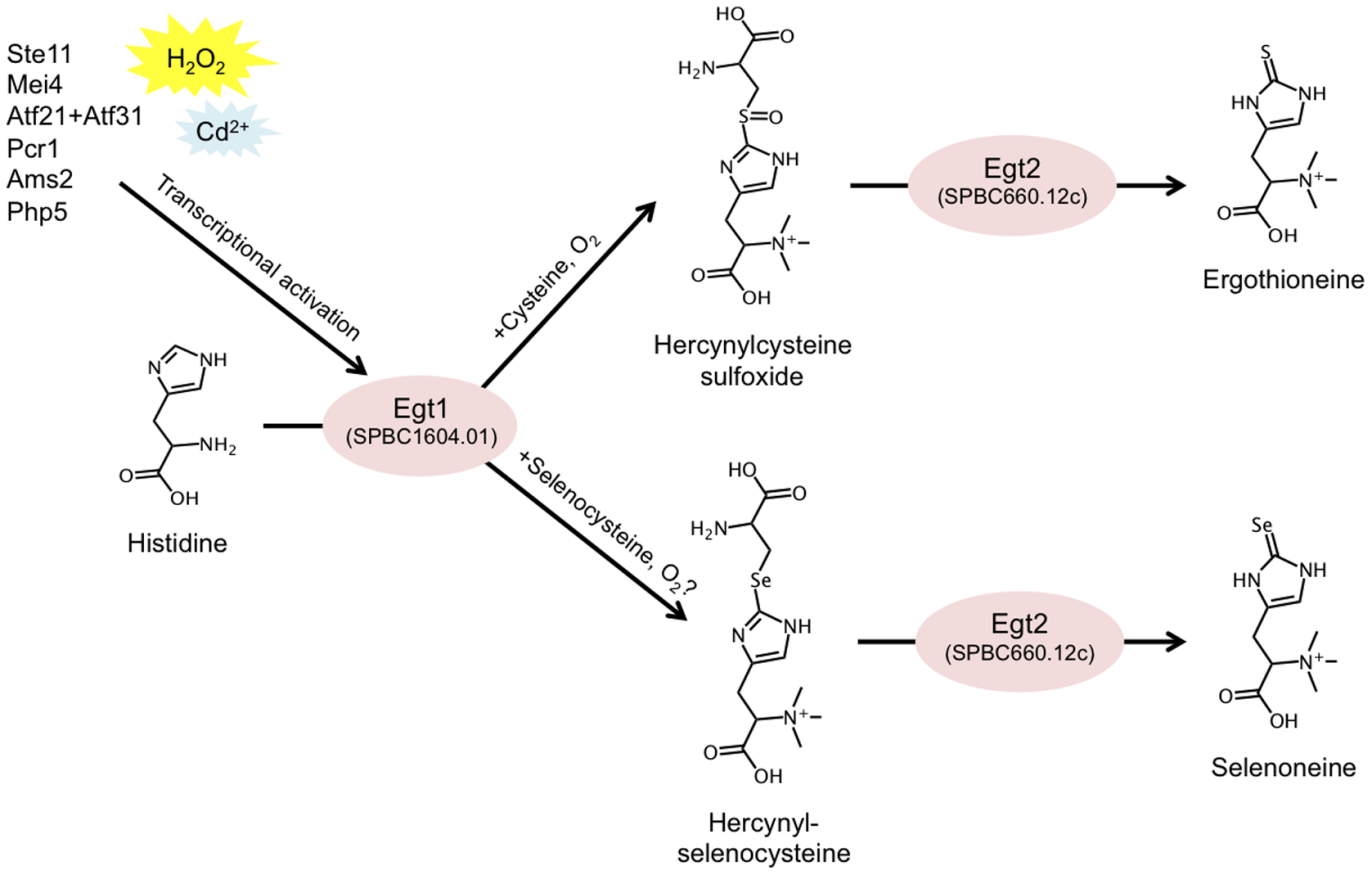
Summary of the described EGT and selenoneine biosynthetic pathway in *S. pombe*, and its transcriptional regulation.

In this study we applied metabolomic analysis to identify the *egt1^+^* and *egt2^+^* genes composing the EGT and selenoneine biosynthetic pathway in *S. pombe* (Figure 4). These two genes have no homologs in *S. cerevisiae*, consistent with the fact that budding yeast do not produce EGT. In the future, a comparison between these two yeasts that appear similar, but are genetically rather distant, might provide useful clues regarding the native physiology of EGT or selenoneine. The presence or absence of the EGT pathway could be related to differences in the ecology of these two species.

In an environmental stress study, transcription of the *egt1^+^* gene was up-regulated ∼10-fold in the presence of 0.5 mM H_2_O_2_, and ∼4-fold in the presence of Cd^2+^ [31]. It thus appears that *egt1^+^* is strongly stress-responsive, providing support for the hypothesized antioxidative role of EGT. In addition, the *egt1^+^* locus was previously named *mug158* ^+^, denoting Meiotically Up-regulated Gene 158, due to its up-regulation upon entry into meiosis [32]. The *egt1^+^* promoter contains the TR-box motif 5′-TTCTTTGTTY-3′, recognized by the sexual development transcription factor, Ste11 [33], and a 5′-GTAAAYA-3′-binding motif recognized by the forkhead transcription factor, Mei4 [34]. Indeed, the *egt1^+^* transcript was up-regulated in strains overexpressing *ste11* ^+^ or *mei4^+^* [35], [36]. Also, *egt1^+^* was up-regulated in a strain overexpressing both basic leucine zipper (bZIP) transcription factors *atf21^+^* and *atf31^+^* [36]. Furthermore, *egt1^+^* was previously reported as a putative regulatory target of the transcription factors, Ams2, Php5, and Pcr1, by analysis of multiple genome-wide microarray datasets [37]. Ams2 is a cell cycle-dependent GATA factor activated during replication [38]. Php5 is a subunit of the CCAAT-binding complex, which regulates respiration, based on glucose and iron availability [39]. Pcr1 is a CREB/ATF protein involved in stress response and sexual development [40], [41]. In addition, the 5′ UTR region of *egt1^+^* contains the CuSE element sequence, 5′-DWDDHGCTGD-3′, which is recognized by the Cuf1 transcription factor and activated by copper deficiency [42]. However, no transcriptional activation of *egt1^+^* was found under varying copper levels [43], suggesting this 5′ UTR DNA fragment might not correspond to an actual Cuf1 binding site. In conclusion, it seems that *egt1^+^* might be under regulatory control of a variety of different transcription factors.

In our study, the Δ*egt1* deletion mutant showed a complete absence of EGT and all its precursors. In the Δ*egt2* deletion mutant, some amount of EGT remained, consistent with results previously reported by Seebeck [12], that hercynylcysteine sulfoxide can spontaneously convert to EGT in the presence of PLP. Judging from the wealth of published transcriptome data, transcription of *egt2^+^* does not vary in response to environmental conditions [31], and is only mildly up-regulated (∼2-fold) during meiosis [32], suggesting that *egt1^+^* represents the main regulatory element in this pathway. The Egt2 enzyme could be ubiquitously present in the cells, simply converting any available hercynylcysteine sulfoxide into EGT.

Overexpression strains, *P81nmt1-egt1^+^*, *P41nmt1-egt1^+^*, and *P3nmt1-egt1^+^*, showed gradual accumulation of EGT in standard EMM2 medium without thiamine, providing an additional confirmation of the correct *egt1^+^* assignment. Interestingly, *P3nmt1-egt1^+^* cells contained a large amount of hercynylcysteine sulfoxide. A possible explanation is that the activity of Egt1 in this strain was higher than the native activity of Egt2, resulting in accumulation of the metabolic intermediate between these two enzymes. These overexpression strains will be invaluable for verification of any proposed EGT mechanism. We also demonstrated that the *P3nmt1-egt1^+^* strain could synthesize a considerable amount of selenoneine when selenium was supplemented in the culture medium. Interestingly, selenoneine biosynthesis, unlike EGT biosynthesis, did not involve a formation of a sulfoxide intermediate, but rather involved a simple conjugate compound, hercynylselenocysteine. It is unknown whether *egt1* ^+^-mediated synthesis of hercynylselenocysteine requires oxygen, as in the case of EGT synthesis. The *P3nmt1-egt1^+^* strain proved to be very useful to elucidate the complete pathway, and we propose that this strain could also be employed industrially to produce EGT, as well as hercynylcysteine sulfoxide, hercynylselenocysteine, or selenoneine (the latter three compounds are not commercially available at present). The signal intensity of selenoneine found in WT nitrogen-starved cells was rather low; however, selenoneine might potentially have an interesting function in *S. pombe*. Selenoneine can be found in humans [44], and it was suggested that its radical-scavenging activity is even higher than that of EGT [30]. Importantly, we showed that selenoneine does not seem to be involved in detoxification of selenium in *S. pombe*.

From a wider perspective, the ubiquitous presence of EGT in living organisms, from bacteria to humans, suggests a crucially important function, yet this function has not been convincingly demonstrated so far. We found only trace amounts (0.3 µM) of EGT in vegetatively growing *S. pombe* cells; however, under starvation-induced quiescence it increased several hundred-fold. That, together with reported up-regulation of the *egt1^+^* transcript in the presence of H_2_O_2_ and in meiosis, implies a supportive or protective role of EGT for long-term hibernation of cells. However, the Δ*egt1* deletion strain did not show any obvious defects in either sporulation or in quiescence. Furthermore, we did not detect any sensitivity of the Δ*egt1* mutant to common oxidants H_2_O_2_ and *tert*-butyl hydroperoxide. As cells contain multiple redundant systems to deal with oxidative stress, it is possible that the lack of EGT could be compensated by another mechanism. On the other hand, the observation that the highly overexpressed *P3nmt1-egt1^+^* strain, which accumulated EGT well beyond normal physiological levels, did not acquire any resistance to the tested oxidants, is intriguing. An attractive possibility exists that the true physiological purpose of EGT might lie in a yet unexplored – and possibly unexpected – area. We propose that genetic and metabolomic analyses, together with the collection of *S. pombe* strains introduced in this manuscript, may provide ideal tools to further investigate the *in vivo* role of this enigmatic compound.

## Materials and Methods

### Amino acid sequence alignment and bioinformatic analysis

Amino acid sequence alignment was performed using the on-line version (http://www.ncbi.nlm.nih.gov/tools/cobalt/) of the Constraint-based Multiple Alignment Tool (COBALT) [45] with default parameters. A phylogenetic tree was generated from the on-line COBALT tool (using the Fast Minimum Evolution method with Max Seq Difference set to 0.9 and other parameters set to default values) and visualized using Analyses of Phylogenetics and Evolution (APE) software [46]. Percentage identity of the amino acid sequences was calculated using the Clustal-Omega algorithm [47] with default parameters.

### Strains and growth conditions

The *S. pombe* strains used in this manuscript are listed in Table 4. The synthetic minimal medium (EMM2), rich yeast extract medium (YE) and sporulation-inducing medium (MEA) recipes were used as published previously [48]. The following variants of the liquid EMM2 medium were used: EMM2-N (EMM2 lacking NH_4_Cl), EMM2-LG (EMM2 containing 1.1 mM – or 0.2 g/l – glucose), EMM2+Se (EMM2 containing additional 10 µM Na_2_SeO_4_), and EMM2-N+Se (EMM2-N containing additional 10 µM Na_2_SeO_4_). Cell cultures were cultivated at 26°C.

**Table 4:** *S. pombe* strains used in this manuscript.

Strain name	Genotype	Source
972	*h^−^* (WT)	Leupold (1950) [52]
975	*h^+^* (WT)	
KS1366	*h^−^* Δ*sty1::ura4^+^ ura4-D18*	Shiozaki and Russell (1995) [53]
TP1701	*h^−^* Δ*nfs1::kanMX4*	Strains from the Bioneer haploid deletion mutant collection [24] were backcrossed with WT 972 to remove auxotrophic markers.
TP1704	*h^−^* Δ*SPBC660.12c::kanMX4*	
TP1705	*h^−^* Δ*SPAC11D3.10::kanMX4*	
TP1706	*h^+^* Δ*SPCC777.03c::kanMX4*	
TP1707	*h^−^* Δ*SPAC11D3.10::hphMX6*	Marker switch of TP1705 to hphMX6
TP1732	*h^−^* Δ*SPBC660.12c::natMX6*	Marker switch of TP1704 to natMX6
TP1733	*h^+^* Δ*SPBC660.12c::natMX6*	TP1732 crossed with WT 975
TP1736	*h^−^* Δ*SPBC660.12c::natMX6 Δnfs1::kanMX4*	TP1701 crossed with TP1733
TP1737	*h^−^* Δ*SPBC660.12c::natMX6 ΔSPCC777.03c::kanMX4*	TP1706 crossed with TP1732
TP1739	*h^+^* Δ*SPBC660.12c::natMX6 ΔSPAC11D3.10::hphMX6*	TP1707 crossed with TP1733
TP1740	*h^−^* Δ*SPCC777.03c::kanMX4 ΔSPAC11D3.10::hphMX6*	TP1706 crossed with TP1707
TP1743	*h^−^* Δ*SPBC660.12c::natMX6 ΔSPAC11D3.10::hphMX6 ΔSPCC777.03c::kanMX4*	TP1739 crossed with TP1740
TP1770	*h^−^* Δ*egt1::kanMX6*	
TP1771	*h^−^* Δ*egt2::kanMX6*	
TP1857	*h^−^ egt1::P81nmt1-egt1^+^*	Constructed as part of this study.
TP1855	*h^−^ egt1::P41nmt1-egt1^+^*	
TP1803	*h^−^ egt1::P3nmt1-egt1^+^*	
TP1813	*h^−^* Δ*egt2::hphMX6*	Marker switch of TP1771 to hphMX6
TP1814	*h^+^* Δ*egt2::hphMX6*	TP1813 crossed with WT 975
TP1879	*h^−^ egt1::P3nmt1-egt1^+^* Δ*egt2::hphMX6*	TP1803 crossed with TP1814

### Construction of mutants

All DNA recombinant strains were constructed using a two-step PCR method. In the first step, two approximately 500-bp regions were amplified using genomic DNA of the WT 972 strain as a template, corresponding to the forward and reverse ends of the recombination cassette. In the second step, both modules were combined with the appropriate plasmid containing the kanamycin resistance marker (kanMX6). Transformants were selected by resistance to geneticin (G418) and correct integrations were verified by PCR. Primer sequences used for all PCR amplifications are included in Table S6.

The gene disruption strains (TP1770 and TP1771) were constructed by replacing the target open reading frames with the kanamycin resistance marker. The *pFA6a-kanMX6* plasmid [26] was used as a template for construction of replacement cassettes. The overexpression strains integrating the *nmt1^+^* promoter (TP1857, TP1855, and TP1803) were constructed using the *pFA6a-kanMX6-P81nmt1, pFA6a-kanMX6-P41nmt1*, and *pFA6a-kanMX6-P3nmt1* plasmids [26] as templates.

Hygromycin-resistant (hphMX6) and clonNAT-resistant (natMX6) versions of the deletion strains (TP1707, TP1732, and TP1813) were prepared by PCR amplification of a marker switch cassette and transformation of the cassette into the original strains, as described previously [49]. The *pAG32* and *pAG25* plasmids [50] were used as templates for the construction of the marker switching cassettes. Crosses were performed by sporulation on an MEA plate followed by tetrad dissection of the formed asci and selection on YE plates containing the appropriate combination of drugs.

### Metabolome sample preparation

Metabolomic analysis was performed as previously described [17]. Briefly, cells from cultures (40 ml/sample, 3.3×10^6^ cells/ml for vegetative cells, or 10^7^ cells/ml for nitrogen-starved cells, respectively) were collected by vacuum filtration and immediately quenched in 25 ml of −40°C methanol. Cells were harvested by centrifugation at −20°C and constant amounts of internal standards (10 nmol of HEPES and PIPES) were added to each sample. Cells were disrupted using a Multi-Beads Shocker (Yasui Kikai, Osaka, Japan). Proteins were removed by filtering on an Amicon Ultra 10-kDa cut-off filter (Millipore, Billerica, USA) and samples were concentrated by vacuum evaporation. Finally, each sample was re-suspended in 40 µl of 50% acetonitrile and 1 µl was used for LC-MS analysis.

### LC-MS analysis

LC-MS data were obtained using a Paradigm MS4 HPLC system (Michrom Bioresources, Auburn, USA) coupled to an LTQ Orbitrap mass spectrometer (Thermo Fisher Scientific, Waltham, USA). LC separation was performed on a ZIC-pHILIC column (Merck SeQuant, Umeå, Sweden; 150×2.1 mm, 5 µm particle size). Acetonitrile (A) and 10 mM ammonium carbonate buffer, pH 9.3 (B) were used as mobile phase, with gradient elution from 80% A to 20% A in 30 min at a flow rate of 100 µl/min. Peak areas of metabolites of interest were measured using the MZmine 2.10 software [51] and normalized by the weighted contribution of the peak areas of the spiked internal standards (HEPES and PIPES). Identification of metabolites reported in this manuscript was based on their theoretical m/z values and MS/MS fragmentation data, with the exception of EGT, the retention time of which was verified by analyzing a pure standard (obtained from Tetrahedron, Vincennes, France). The identity of selenium-containing compounds was verified by their isotope distribution patterns (Figure S10).

### Spot test assays

Plate media were prepared using standard EMM2 recipe [48] with 17 g/l agar. Reagents were added to the media after autoclaving to final concentrations as indicated. Cells were cultivated to a concentration of 5×10^6^ cells/ml and serially diluted in 6 steps (5-fold dilution in each step). 5 µl of the diluted cultures was plated in each spot. Plates were incubated at 26°C for 6 days.

### Viability measurement

Cell viability was measured by plating approximately 300 cells on a YE agar plate, incubating the plate at 26°C for 4–5 days, and counting the number of colonies formed. Viability was calculated as the percentage of the number of formed colonies against the number of colonies formed at the first time point.

## Supporting Information

Figure S1 **Amino acid sequence alignment of** ***S. pombe*** **SPBC1604.01,** ***S. japonicus*** **SJAG_00832,** ***N. crassa*** **Egt-1,** ***M. smegmatis*** **EgtD, and** ***M. smegmatis*** **EgtB proteins.** Alignment was generated using the COBALT algorithm. Conserved domains are indicated according to their location in *S. pombe* SPBC1604.01.
(TIF)

Figure S2 **Amino acid sequence alignment of** ***M. smegmatis*** **EgtE protein and its four putative homologs in** ***S. pombe*** **.** Alignment was generated using the COBALT algorithm. The conserved catalytic residue (PLP binding site) is indicated by a red arrow.
(TIF)

Figure S3 **Normalized peak areas of EGT and its precursors obtained by metabolomic analysis of WT, the** **Δ** ***SPBC660.12c*** **single deletion mutant, and multiple deletion mutants with other putative homologs of mycobacterial EgtE.** Cells were nitrogen-starved prior to analysis (24 h in EMM2-N medium) to induce EGT synthesis.
(TIF)

Figure S4 **Amino acid sequence alignment of** ***S. pombe*** **SPBC1604.01 (Egt1) protein and its closest homologs in selected species.** Alignment was generated using the COBALT algorithm. Conserved domains are indicated according to their location in *S. pombe* Egt1.
(TIF)

Figure S5 **Amino acid sequence alignment of** ***S. pombe*** **SPBC660.12c (Egt2) protein and its closest homologs in other species.** Alignment was generated using the COBALT algorithm. Conserved domains are indicated according to their location in *S. pombe* Egt2. The conserved catalytic residue (PLP binding site) is indicated by a red arrow.
(TIF)

Figure S6 **Absolute quantification of EGT content in cells.** A calibration curve was constructed by performing LC-MS injections of pure ergothioneine in 10-fold dilution steps, containing a constant amount of HEPES and PIPES standards (250 pmol each) for normalization (upper panel). Normalized peak areas were plotted against injected amounts and a regression curve was generated using Microsoft Excel. The formula to calculate absolute amount (fmol) from normalized peak area was derived from the regression curve formula (middle panel). Normalized peak areas of EGT were converted into absolute intracellular concentrations using estimated average cellular volumes (bottom panel).
(TIF)

Figure S7 **Spot test results on hydrogen peroxide and** ***tert*** **-butyl hydroperoxide agar plates.** WT, deletion, and overexpression strains described in this manuscript were serially diluted and grown on EMM2 plates supplemented with increasing concentrations of oxidants hydrogen peroxide (H_2_O_2_) and *tert*-butyl hydroperoxide (*t*-BOOH). The stress-sensitive Δ*sty1* strain was used as a positive control.
(TIF)

Figure S8 **Cell number increase in liquid EMM2 medium supplemented with Na_2_SeO_4_.** Relative cell number increase in 24 h was measured in liquid EMM2 medium supplemented with increasing concentrations of Na_2_SeO_4_. Cell cultures were incubated at 26°C.
(TIF)

Figure S9 **Analysis of a mixture of EGT and selenium** ***in vitro*** **.** Extracted ion chromatograms of EGT and selenoneine masses are shown for 1 mM EGT, 1 mM Na_2_SeO_4_, and mixture of both, incubated at room temperature for 24 h. Note that the intensity scale of the selenoneine plot is 0.1% relative to that of the EGT plot.
(TIF)

Figure S10 **Verification of the identity of selenoneine and hercynylselenocysteine by their isotopic patterns.** Comparison of detected vs. calculated isotope distribution patterns of selenoneine (A) and hercynylselenocysteine (B). Theoretical isotope patterns were generated from the corresponding chemical formulas using the Xcalibur software (Thermo Fisher Scientific, Waltham, USA).
(TIF)

Figure S11 **Spot test results on Na_2_SeO_4_ agar plates.** WT, deletion, and overexpression strains described in this manuscript were serially diluted and grown on EMM2 plates supplemented with increasing concentrations of Na_2_SeO_4_. The stress-sensitive Δ*sty1* strain was used as a positive control.
(TIF)

Table S1 **Locations and amino acid sequences of conserved protein domains shown in** **Figure 1B** **and Figure S1.**
(XLSX)

Table S2 **Locations and amino acid sequences of conserved protein domains shown in** **Figure 1C** **and Figure S2.**
(XLSX)

Table S3 **Results of all LC-MS measurements of the intermediates in the EGT biosynthetic pathway.**
(XLSX)

Table S4 **Locations and amino acid sequences of conserved protein domains of Egt1 homologs shown in** **Table 2** **and Figure S4.**
(XLSX)

Table S5 **Locations and amino acid sequences of conserved protein domains of Egt2 homologs shown in** **Table 2** **and Figure S5.**
(XLSX)

Table S6 **Sequences of oligonucleotide primers used for PCR amplifications.**
(XLSX)
