# Correction: Genetic and Metabolomic Dissection of the Ergothioneine and Selenoneine Biosynthetic Pathway in the Fission Yeast, *S. pombe*, and Construction of an Overproduction System

**DOI:** 10.1371/journal.pone.0105177

**Published:** 2014-07-31

**Authors:** The PLOS ONE Staff

There are formatting errors in Tables 1, 3, and 4 of the published article. The correct tables can be viewed here.

**Table 1:** **Normalized peak areas of the four compounds composing the EGT biosynthetic pathway obtained by metabolomic analysis of WT and newly constructed strains.** Values were measured from metabolome samples of three different *S. pombe* strains in three different cultivation conditions, as indicated. Mass values (m/z) and LC retention times (min) of each peak are included for reference.

Strain	Cultivation condition	Histidine, 156.077 m/z @12.4 min	Trimethyl histidine (hercynine), 198.124 m/z @10.3 min	Hercynylcysteine sulfoxide, 333.123 m/z @12.2 min	Ergothioneine, 230.096 m/z @12.6 min
WT 972	EMM2	14.4	1.7	0	0.1
WT 972	EMM2-N (24 h)	2.2	3.2	2.2	13.7
WT 972	EMM2-LG (24 h)	55.4	65.9	3.5	6.7
*Δegt1*	EMM2	10.7	0.3	0	0
*Δegt1*	EMM2-N (24 h)	2.8	0.1	0	0
*Δegt1*	EMM2-LG (24 h)	54.1	0	0	0
*Δegt2*	EMM2	11.4	0.9	3.9	0
*Δegt2*	EMM2-N (24 h)	1.9	1.8	58.2	3.1
*Δegt2*	EMM2-LG (24 h)	61.1	64.7	44.1	1.6

**Table 3:** **Absolute intracellular EGT concentrations (µM) in *S. pombe* cells.** Intracellular concentrations were derived from measured normalized peak areas using a calibration curve generated by injections of pure EGT in 10-fold dilution steps. The detailed calculation method is described in Figure S6.

Cell condition	Culture medium	Intracellular EGT (µM)
WT vegetative	EMM2	0.3
WT nitrogen starvation	EMM2-N (24 h)	157.4
WT glucose starvation	EMM2-LG (24 h)	41.6
*P81nmt1-egt1^+^*	EMM2	32.4
*P41nmt1-egt1^+^*	EMM2	181.2
*P3nmt1-egt1^+^*	EMM2	1606.3

**Table 4:** ***S. pombe* strains used in this manuscript. **Strains from the Bioneer haploid deletion mutant collection [24] were backcrossed with WT 972 to remove auxotrophic markers.

Strain name	Genotype	Source
972	*h^-^* (WT)	Leupold (1950) [52]
975	*h^+^* (WT)	Leupold (1950) [52]
KS1366	*h^-^* Δ*sty1::ura4^+^ ura4-D18*	Shiozaki and Russell (1995) [53]
TP1701	*h^-^ Δnfs1::kanMX4*	Bioneer collection [24]
TP1704	*h^-^ ΔSPBC660.12c::kanMX4*	Bioneer collection [24]
TP1705	*h^-^ ΔSPAC11D3.10::kanMX4*	Bioneer collection [24]
TP1706	*h^+^ ΔSPCC777.03c::kanMX4*	Bioneer collection [24]
TP1707	*h^-^ ΔSPAC11D3.10::hphMX6*	Marker switch of TP1705 to hphMX6
TP1732	*h^-^ ΔSPBC660.12c::natMX6*	Marker switch of TP1704 to natMX6
TP1733	*h^+^ ΔSPBC660.12c::natMX6*	TP1732 crossed with WT 975
TP1736	*h^-^ ΔSPBC660.12c::natMX6 Δnfs1::kanMX4*	TP1701 crossed with TP1733
TP1737	*h^-^ ΔSPBC660.12c::natMX6 ΔSPCC777.03c::kanMX4*	TP1706 crossed with TP1732
TP1739	*h^+^ ΔSPBC660.12c::natMX6 ΔSPAC11D3.10::hphMX6*	TP1707 crossed with TP1733
TP1740	*h^-^ ΔSPCC777.03c::kanMX4 ΔSPAC11D3.10::hphMX6*	TP1706 crossed with TP1707
TP1743	*h^-^ ΔSPBC660.12c::natMX6 ΔSPAC11D3.10::hphMX6 ΔSPCC777.03c::kanMX4*	TP1739 crossed with TP1740
TP1770	*h^-^ Δegt1::kanMX6*	Constructed as part of this study
TP1771	*h^-^ Δegt2::kanMX6*	Constructed as part of this study
TP1857	*h^-^ egt1::P81nmt1-egt1^+^*	Constructed as part of this study
TP1855	*h^-^ egt1::P41nmt1-egt1^+^*	Constructed as part of this study
TP1803	*h^-^ egt1::P3nmt1-egt1^+^*	Constructed as part of this study
TP1813	*h^-^ Δegt2::hphMX6*	Marker switch of TP1771 to hphMX6
TP1814	*h^+^ Δegt2::hphMX6*	TP1813 crossed with WT 975
TP1879	*h^-^ egt1::P3nmt1-egt1^+^ Δegt2::hphMX6*	TP1803 crossed with TP1814
