# Supplementary figures and images for: Genetic and Metabolomic Dissection of the Ergothioneine and Selenoneine Biosynthetic Pathway in the Fission Yeast, S. pombe, and Construction of an Overproduction System
Source: PLoS One. 2014 May 14;9(5):e97774. doi: 10.1371/journal.pone.0097774 (PMC4020840; doi:10.1371/journal.pone.0097774)

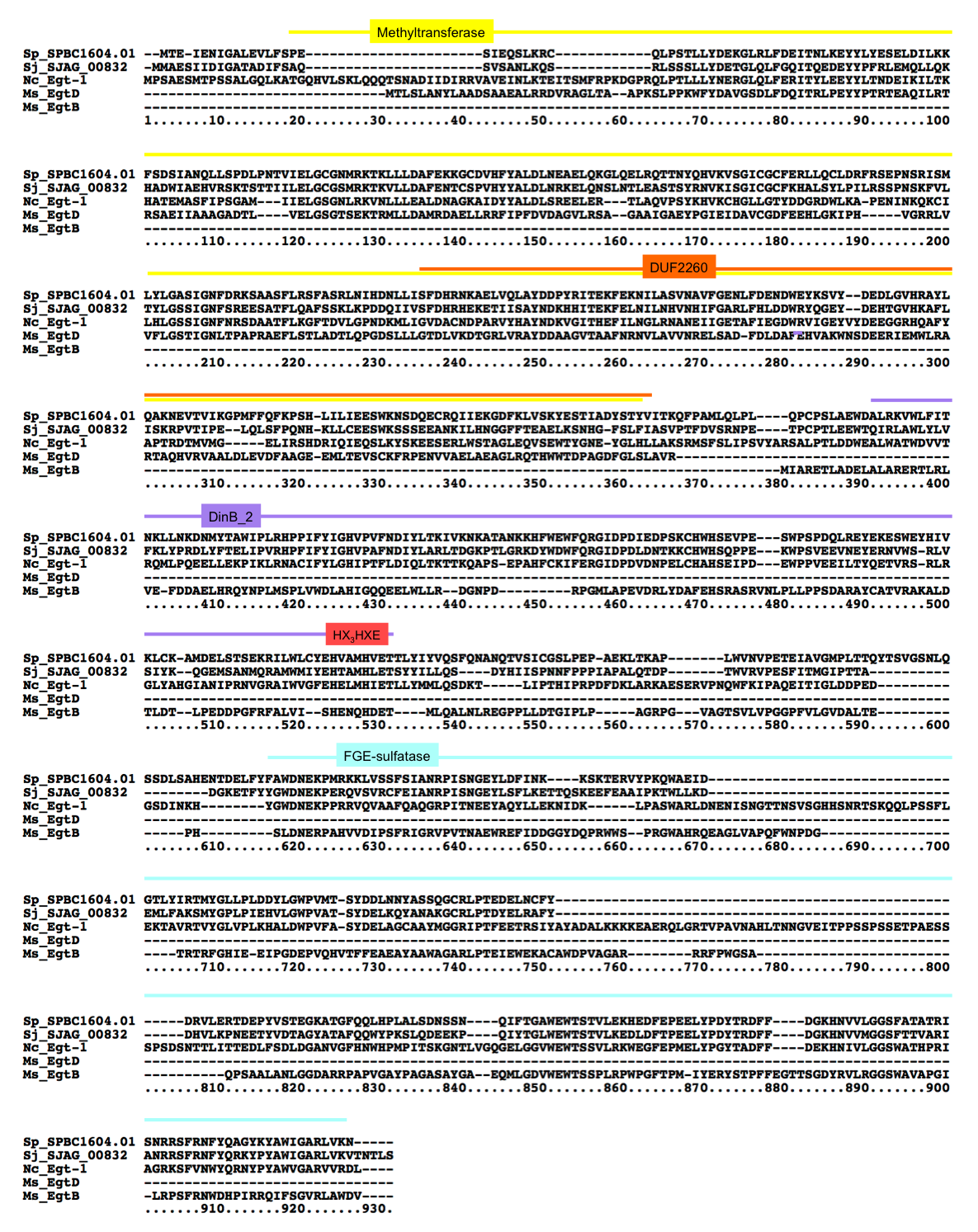

Supplement: Figure S1 — Amino acid sequence alignment of S. pombe SPBC1604.01, S. japonicus SJAG_00832, N. crassa Egt-1, M. smegmatis EgtD, and M. smegmatis EgtB proteins. Alignment was generated using the COBALT algorithm. Conserved domains are indicated according to their location in S. pombe SPBC1604.01. (TIF) [file pone.0097774.s001.tif]

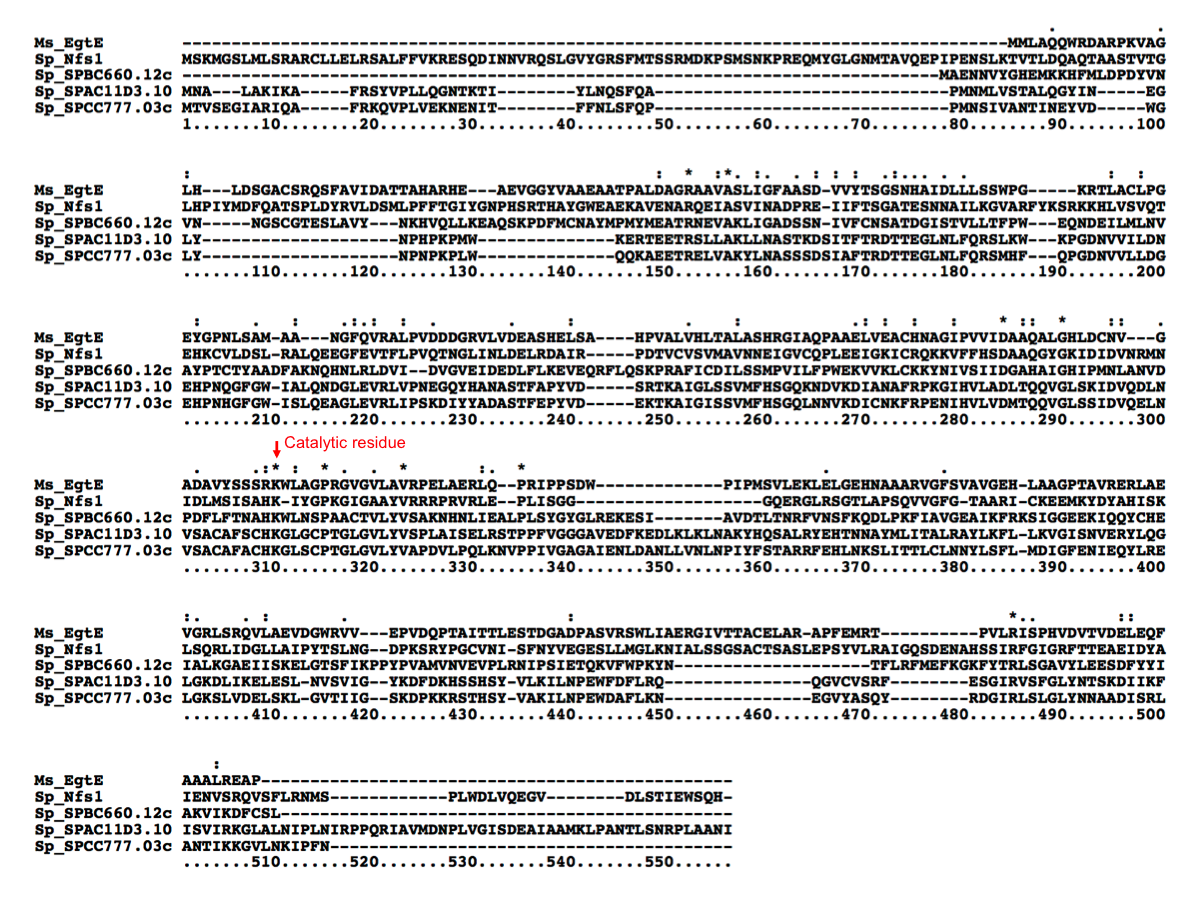

Supplement: Figure S2 — Amino acid sequence alignment of M. smegmatis EgtE protein and its four putative homologs in S. pombe . Alignment was generated using the COBALT algorithm. The conserved catalytic residue (PLP binding site) is indicated by a red arrow. (TIF) [file pone.0097774.s002.tif]

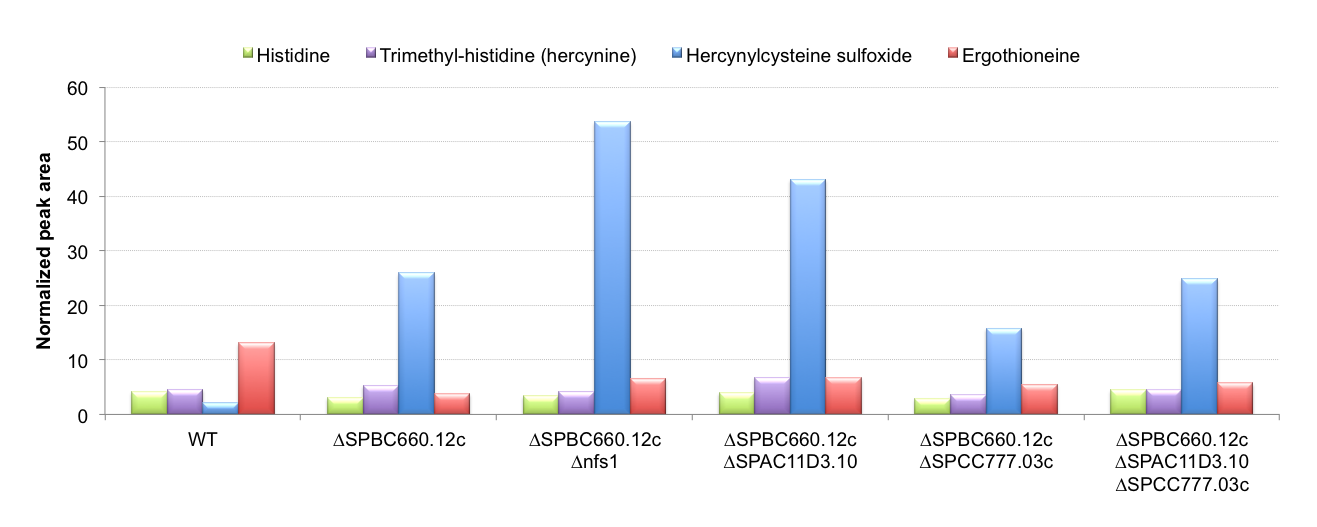

Supplement: Figure S3 — Normalized peak areas of EGT and its precursors obtained by metabolomic analysis of WT, the Δ SPBC660.12c single deletion mutant, and multiple deletion mutants with other putative homologs of mycobacterial EgtE. Cells were nitrogen-starved prior to analysis (24 h in EMM2-N medium) to induce EGT synthesis. (TIF) [file pone.0097774.s003.tif]

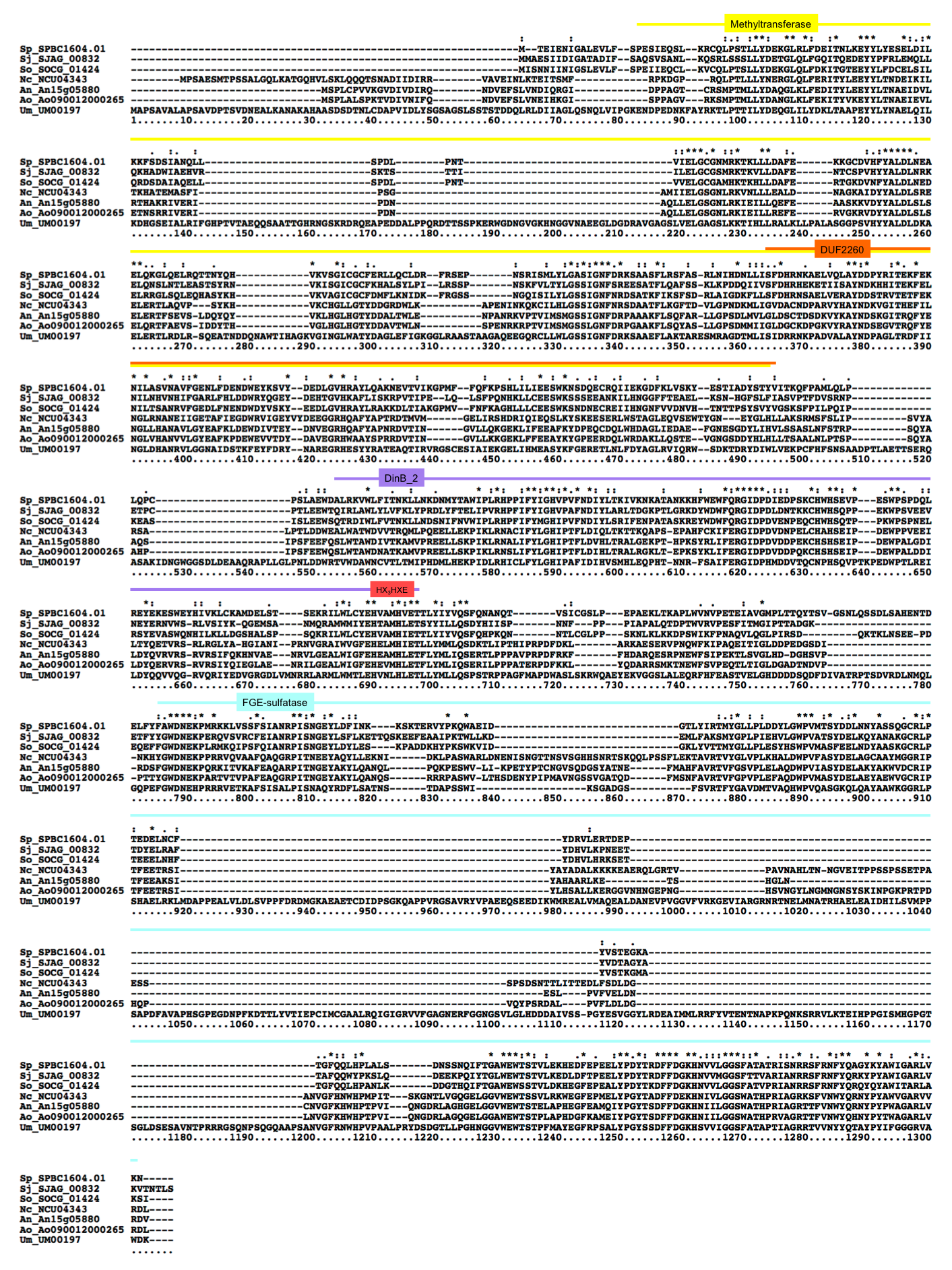

Supplement: Figure S4 — Amino acid sequence alignment of S. pombe SPBC1604.01 (Egt1) protein and its closest homologs in selected species. Alignment was generated using the COBALT algorithm. Conserved domains are indicated according to their location in S. pombe Egt1. (TIF) [file pone.0097774.s004.tif]

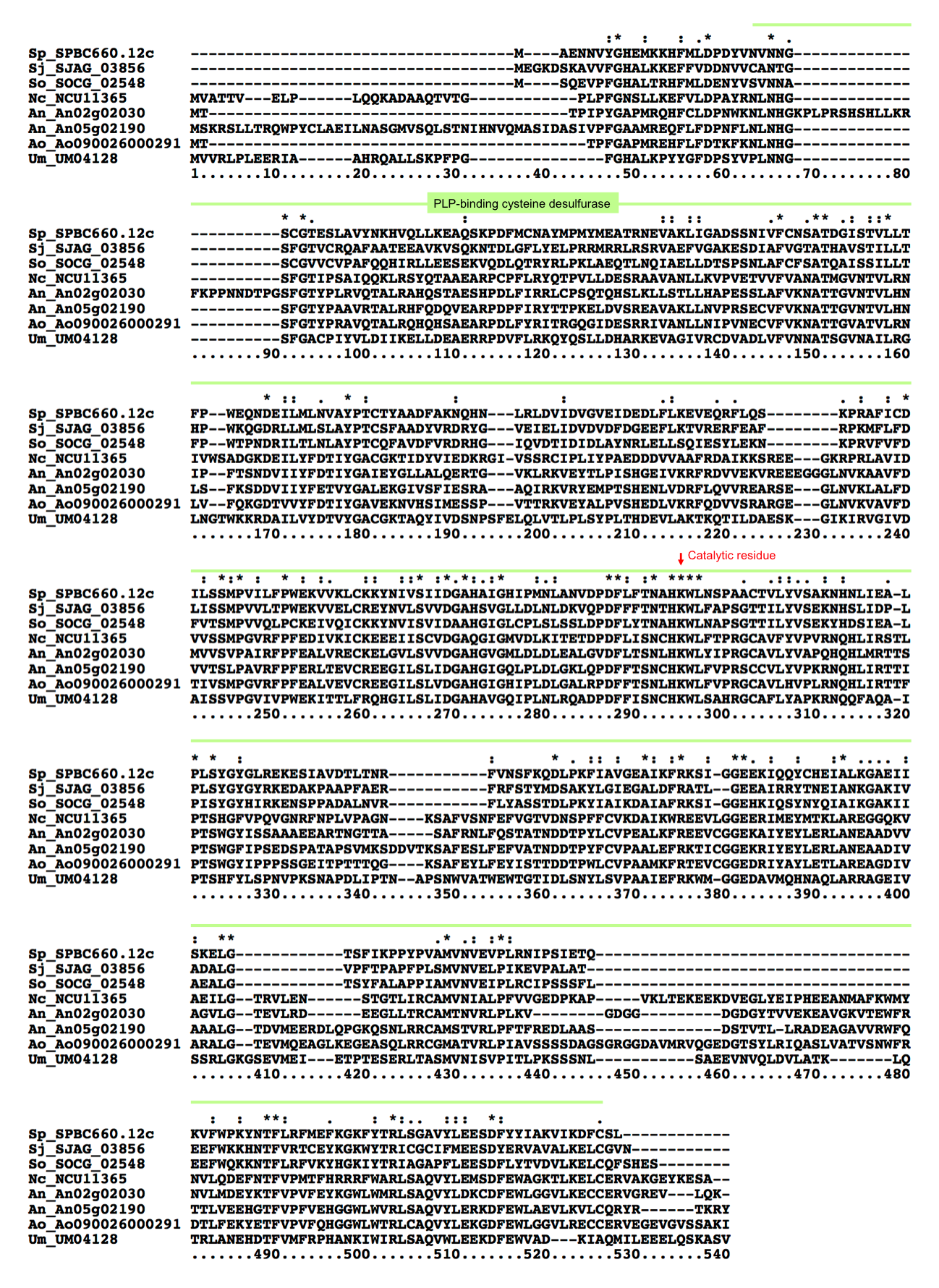

Supplement: Figure S5 — Amino acid sequence alignment of S. pombe SPBC660.12c (Egt2) protein and its closest homologs in other species. Alignment was generated using the COBALT algorithm. Conserved domains are indicated according to their location in S. pombe Egt2. The conserved catalytic residue (PLP binding site) is indicated by a red arrow. (TIF) [file pone.0097774.s005.tif]

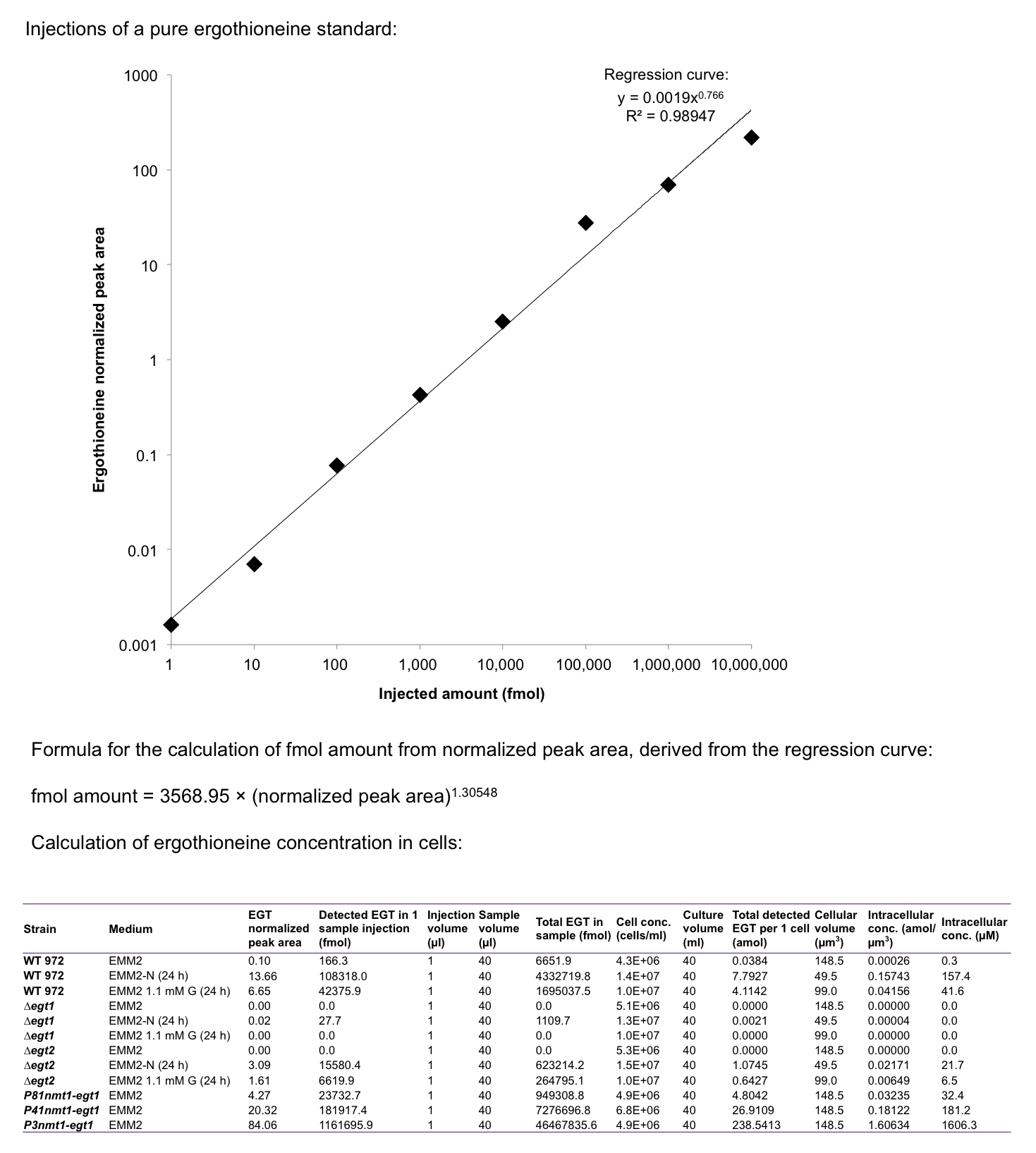

Supplement: Figure S6 — Absolute quantification of EGT content in cells. A calibration curve was constructed by performing LC-MS injections of pure ergothioneine in 10-fold dilution steps, containing a constant amount of HEPES and PIPES standards (250 pmol each) for normalization (upper panel). Normalized peak areas were plotted against injected amounts and a regression curve was generated using Microsoft Excel. The formula to calculate absolute amount (fmol) from normalized peak area was derived from the regression curve formula (middle panel). Normalized peak areas of EGT were converted into absolute intracellular concentrations using estimated average cellular volumes (bottom panel). (TIF) [file pone.0097774.s006.tif]

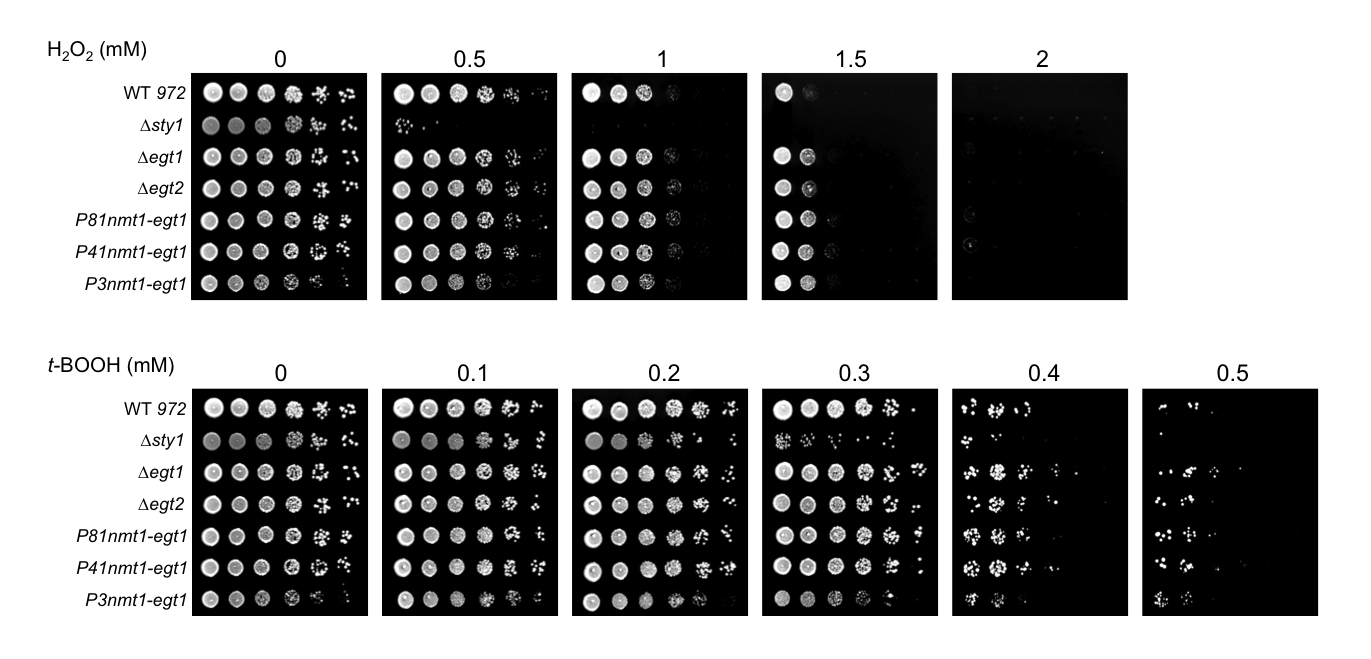

Supplement: Figure S7 — Spot test results on hydrogen peroxide and tert -butyl hydroperoxide agar plates. WT, deletion, and overexpression strains described in this manuscript were serially diluted and grown on EMM2 plates supplemented with increasing concentrations of oxidants hydrogen peroxide (H2O2) and tert-butyl hydroperoxide (t-BOOH). The stress-sensitive Δsty1 strain was used as a positive control. (TIF) [file pone.0097774.s007.tif]

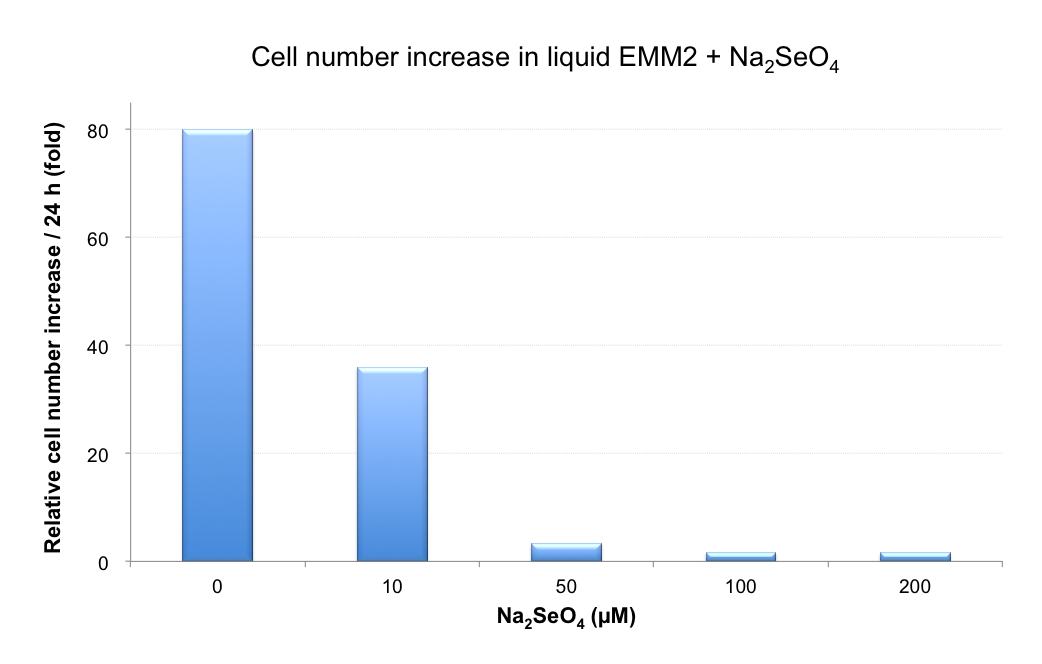

Supplement: Figure S8 — Cell number increase in liquid EMM2 medium supplemented with Na2SeO4. Relative cell number increase in 24 h was measured in liquid EMM2 medium supplemented with increasing concentrations of Na2SeO4. Cell cultures were incubated at 26°C. (TIF) [file pone.0097774.s008.tif]

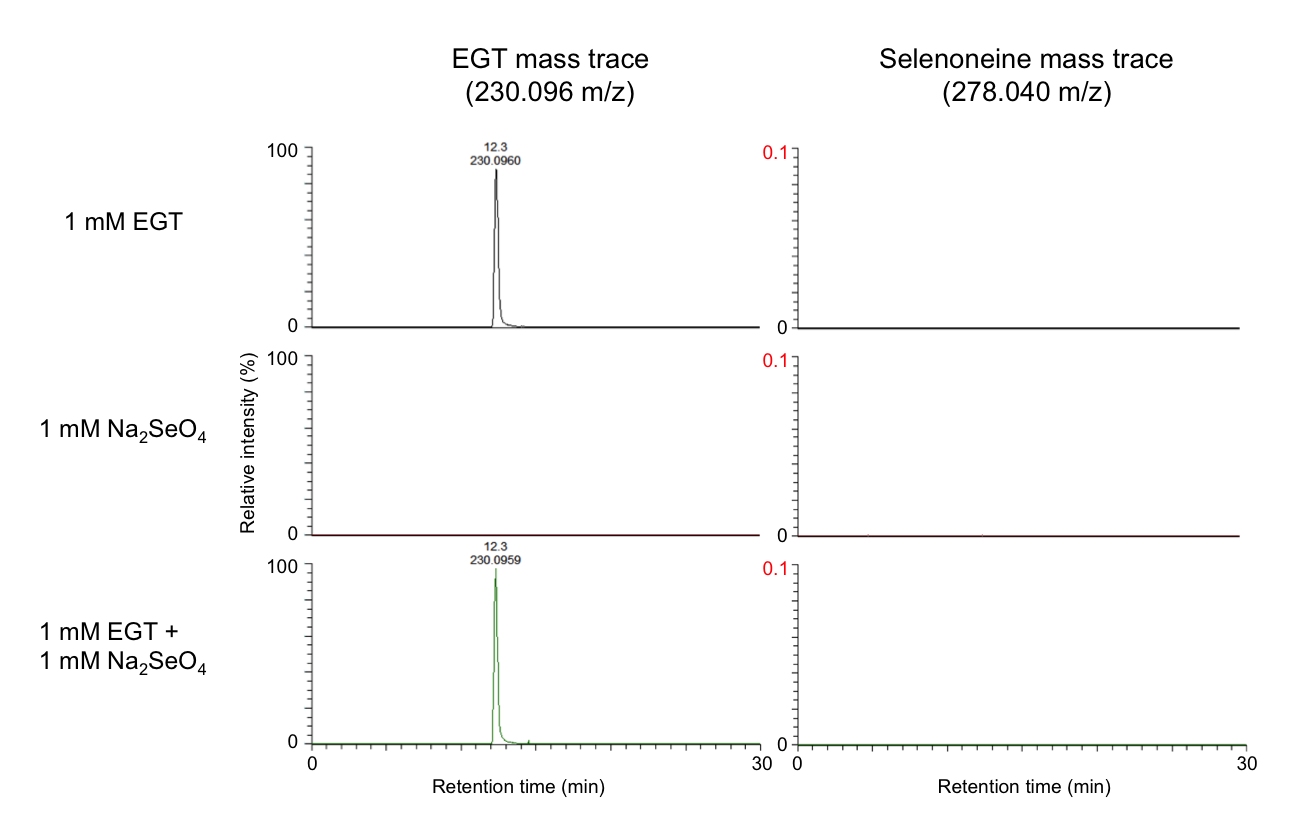

Supplement: Figure S9 — Analysis of a mixture of EGT and selenium in vitro . Extracted ion chromatograms of EGT and selenoneine masses are shown for 1 mM EGT, 1 mM Na2SeO4, and mixture of both, incubated at room temperature for 24 h. Note that the intensity scale of the selenoneine plot is 0.1% relative to that of the EGT plot. (TIF) [file pone.0097774.s009.tif]

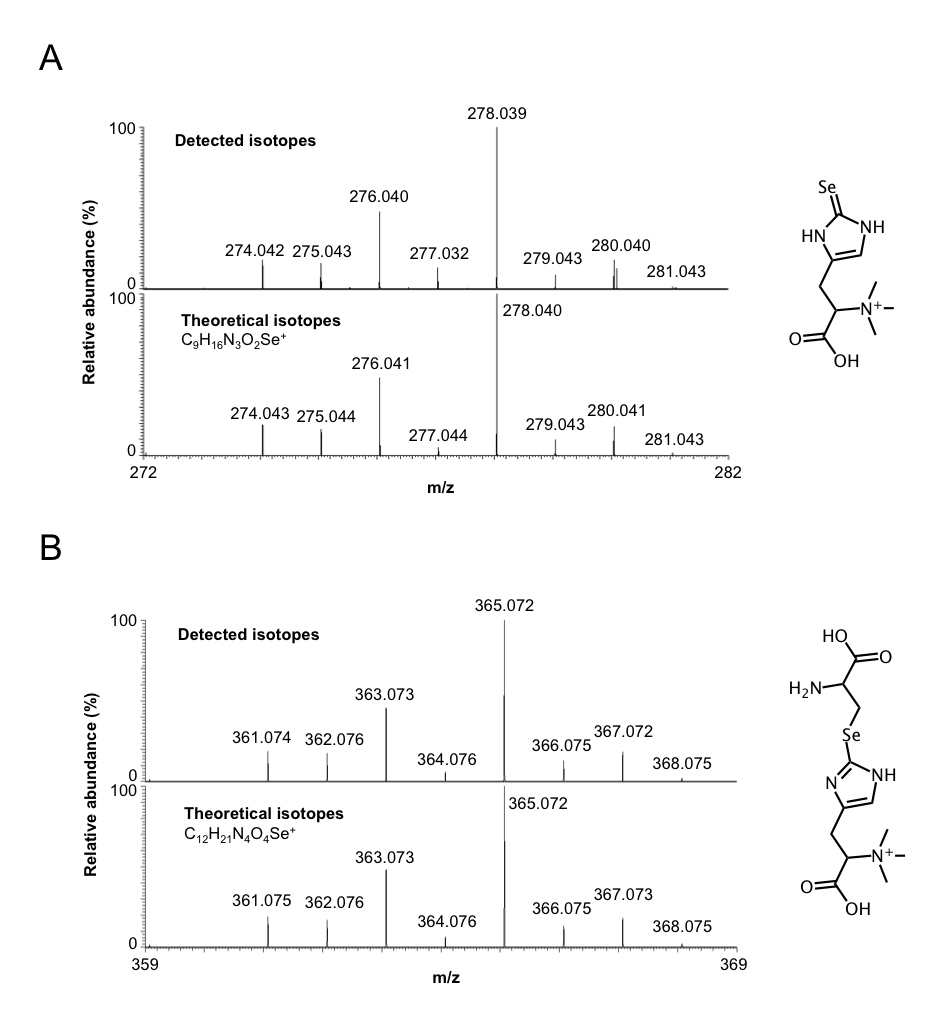

Supplement: Figure S10 — Verification of the identity of selenoneine and hercynylselenocysteine by their isotopic patterns. Comparison of detected vs. calculated isotope distribution patterns of selenoneine (A) and hercynylselenocysteine (B). Theoretical isotope patterns were generated from the corresponding chemical formulas using the Xcalibur software (Thermo Fisher Scientific, Waltham, USA). (TIF) [file pone.0097774.s010.tif]

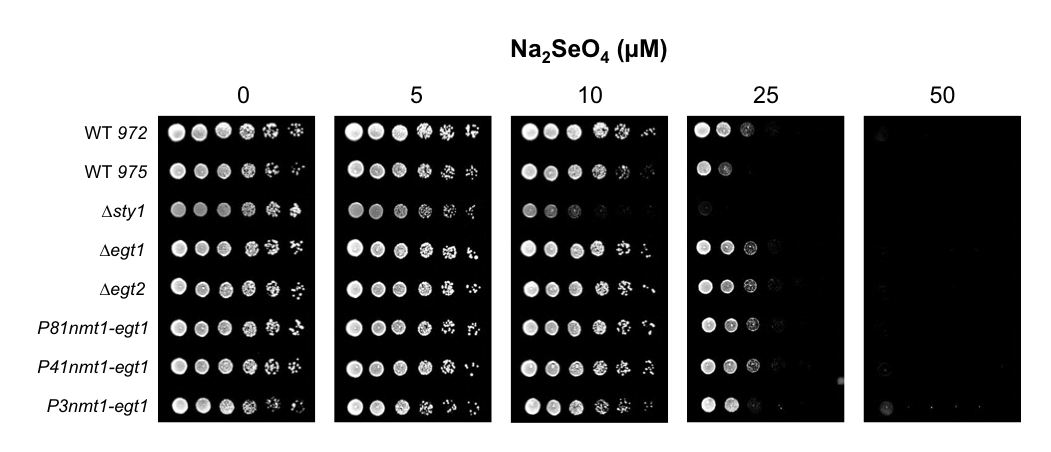

Supplement: Figure S11 — Spot test results on Na2SeO4 agar plates. WT, deletion, and overexpression strains described in this manuscript were serially diluted and grown on EMM2 plates supplemented with increasing concentrations of Na2SeO4. The stress-sensitive Δsty1 strain was used as a positive control. (TIF) [file pone.0097774.s011.tif]
